# Low pelvic incidence with low lordosis and distal apex of lumbar lordosis associated with higher rates of abnormal spinopelvic mobility in patients undergoing THA

**DOI:** 10.1302/2633-1462.49.BJO-2023-0091.R1

**Published:** 2023-09-03

**Authors:** Thomas Aubert, Philippe Gerard, Guillaume Auberger, Guillaume Rigoulot, Guillaume Riouallon

**Affiliations:** 1 Department of Orthopaedic Surgery, Deaconess Saint Simon Cross Hospital Group, Paris, Île-de-France, France; 2 Department of Orthopaedic Surgery, Clinique Arago, Paris, France; 3 Orthopaedic department, Paris Saint Joseph Hospital Group, Paris, Île-de-France, France

**Keywords:** Spine-hip relationship, hip arthroplasty, spinopelvic mobility, pelvic incidence, lumbar shape, lumbar lordosis, lordosis, spine, spinopelvic tilt, pelvis, Multivariate analysis, Total hip arthroplasty (THA), hip

## Abstract

**Aims:**

The risk factors for abnormal spinopelvic mobility (SPM), defined as an anterior rotation of the spinopelvic tilt (∆SPT) ≥ 20° in a flexed-seated position, have been described. The implication of pelvic incidence (PI) is unclear, and the concept of lumbar lordosis (LL) based on anatomical limits may be erroneous. The distribution of LL, including a unusual shape in patients with a high lordosis, a low pelvic incidence, and an anteverted pelvis seems more relevant.

**Methods:**

The clinical data of 311 consecutive patients who underwent total hip arthroplasty was retrospectively analyzed. We analyzed the different types of lumbar shapes that can present in patients to identify their potential associations with abnormal pelvic mobility, and we analyzed the potential risk factors associated with a ∆SPT ≥ 20° in the overall population.

**Results:**

ΔSPT ≥ 20° rates were 28.3%, 11.8%, and 14.3% for patients whose spine shape was low PI/low lordosis (group 1), low PI anteverted (group 2), and high PI/high lordosis (group 3), respectively (p = 0.034). There was no association between ΔSPT ≥ 20° and PI ≤ 41° (odds ratio (OR) 2.01 (95% confidence interval (CI)0.88 to 4.62), p = 0.136). In the multivariate analysis, the following independent predictors of ΔSPT ≥ 20° were identified: SPT ≤ -10° (OR 3.49 (95% CI 1.59 to 7.66), p = 0.002), IP-LL ≥ 20 (OR 4.38 (95% CI 1.16 to 16.48), p = 0.029), and group 1 (OR 2.47 (95% CI 1.19; to 5.09), p = 0.0148).

**Conclusion:**

If the PI value alone is not indicative of SPM, patients with a low PI, low lordosis and a lumbar apex at L4-L5 or below will have higher rates of abnormal SPM than patients with a low PI anteverted and high lordosis.

Cite this article: *Bone Jt Open* 2023;4(9):668–675.

## Introduction

Total hip arthroplasty (THA) can be a very successful surgery,^1^ even if a small percentage of patients who undergo THA have a higher rate of complications, such as impingement, instability, dislocation, and need for early revision.^2,3^ Abnormal lumbopelvic cinematic can lead to aberrant functional acetabular orientation. For this reason, the spine hip relationship (SHR) has been described to better understand the close relationship between the spine and the hip,^4^ and to identify the risk factors for abnormal spinopelvic mobility,^5^ defined as an anterior rotation of the spinopelvic tilt (SPT) ≥ 20° from a standing to a sitting position (standing SPT≤-10°, sagittal spinal deformity (SSD; pelvic incidence (PI) lumbar lordosis (LL) mismatch ≥ 20°), and stiffness of the spine (lumbar flexion (LF) ≤ 20°).^6^ This explains the necessity of assessing biomechanical parameters preoperatively using preoperative imaging (dynamic spinopelvic radiographs) to make an appropriate plan and to select the best implants.^5^

However, all patients who present with abnormal mobility of the pelvis do not have these risk factors,^7^ and some authors attribute a higher risk of anterior impingement in patients with a low lordosis without degenerative spine,^8-10^ explained by a stiffer lumbo-pelvic complex, describing them as “hip users”.^4^ The PI being directly related to lumbar lordosis (lower in patients with low lordosis and higher in patients with high lordosis),^11^ some authors consider a relation between the PI and abnormal pelvic mobility.^9,10^ However, recent studies have shown that the risk of having an abnormal spinopelvic mobility in patients with a PI < 41° is not increased by analyzing more than 9,000 consecutive patients,^7^ and that the risk of dislocation following THA with the same boundary is not increased,^6^ thereby concluding that the PI value alone may not be a risk factor for THA instability.

Roussouly et al^11^ reported the concept of LL based on anatomical limits as erroneous, thereby emphasizing the importance of considering the distribution of LL and classified patients without SSD (PI-LL > 10°) into four types of patients,^11^ according to the curve and apex of LL, pelvic incidence, and sacral slope. Consequently, a global analysis of the spinopelvic parameters could more effectively identify patients without SSD, stiff spine, or excessive posterior spinopelvic tilt, but with a high risk of having abnormal spinopelvic mobility. Furthermore, a new type of lordosis was described; type 3 AP (anteverted pelvis) with an unusual and new spine shape, accounting for 16% of the healthy population that has a low-grade PI but an SS > 35°, a low posterior or anterior SPT, and slight hyperlodosis with a lumbar apex at the L4 level.^12^ Patients presenting this new shape with a low PI have the same biomechanics as patients with a higher PI and also differ from the classic description of patients with low PI, which could explain the debate surrounding the implication of PI in either spinal or pelvic mobility.

The objectives of this study were to analyze the different types of lumbar shape in patients and their different spinopelvic parameters in a cohort of patients who underwent THA, and to analyze the risk factors associated with a ∆SPT ≥ 20° in this overall population, including the types of lumbar shape.

## Methods

### Study design and participants

A retrospective series of 311 consecutive patients who underwent primary THA and had available lateral functional radiographs and low-dose CT scans taken between January 2020 and March 2023 were included. Preoperative planning using the Optimized Positioning System (OPSInsight; Corin, UK) was implemented for cementless THA with ceramic-on-ceramic bearings (Meije Dynacup; Corin). The mean age of the patients was 63.8 years (24 to 82 years), with 182 females and 129 males. This study was approved by the local ethics committee.

Two lateral X-rays were taken for each patient between three months and six weeks before surgery, one of the upper body, standing in a relaxed posture with their feet shoulder-width apart, and one in a flexed-seated position, with their femora parallel to the floor.

### Spinopelvic and pelvic mobility parameters

The measurements taken on the lateral X-rays were SS, standing and flexed-seated LL, and standing and flexed-seated SPT.^13^ Anterior rotation of SPT was assigned a positive value, and posterior rotation of SPT was assigned a negative value. An increase in SPT denotes an anterior rotation of the pelvis that is equivalent to anteversion, which decreases PT. The measurements taken from the bony landmarks on CT scan were the PI.

We investigated the PI-LL mismatch, defined as the difference between PI and LL in the standing position, and the LF, defined as the difference between standing and flexed-seated LL.

The parameters included pelvic mobility during transition from the standing to sitting position, measured as the difference between standing and flexed-seated SPT (∆SPT). All imaging findings were analyzed by two independent engineers.^14^

Two surgeons (TA, PG) measured the pelvic femoral angle (PFA), which is the angle that is formed by making a line from the centre of the S1 end plate to the centre of the femoral head and making a second line that is parallel to the femoral diaphysis.^9^ The lumbar apex was analyzed by using the Roussouly classification.^11^ Femur mobility was measured as the difference between standing and flexed-seated PFA (∆PFA).

### Outcome

We divided the population in terms of LL shape into three groups:

- Group 1: Roussouly types 1 and 2 (PI ≤ 45°, low lordosis, and lumbar apex at disk level L4-L5 or below).
- Group 2: Roussouly type 3 anteverted (low PI ≤ 45° anteverted, low lordosis, and lumbar apex at level L3).
- Group 3: Roussouly types 3 and 4 (PI > 45° high lordosis with a lumbar apex at level L3 or L4).

Because an arthritic hip can be stiff with compensative anteversion of the pelvis,^15^ and the accuracy of PI on CT scan is higher,^16^ we decided to identify the different groups of patients by evaluating only the PI and lumbar apex.

The outcome of interest was abnormal spinopelvic mobility, defined as ΔSPT ≥ 20° between the standing and relaxed-sitting positions.^7^ We analyzed SPT≤-10°, LF ≤ 20°, PI-LL ≥ 20, PI ≤ 41°, SS < 35°, and the different groups of lordosis as risk factors in the overall population.

### Statistical analysis

Continuous variables are described as means and ranges. Continuous outcomes were compared with analysis of variance (ANOVA), Welch ANOVA, or Kruskal-Wallis tests according to data distribution. Discrete outcomes were compared with chi-squared or Fisher’s exact test accordingly. The associations between ΔSPT ≥ 20° and the spinopelvic parameters were assessed using logistic regression analyses. There was no missing data. The factors identified in the univariate analysis were selected for multivariable analysis if the p-value of the Wald test < 0.200. The parameters of the final, multivariable model were identified using a stepwise backward elimination until each parameter was associated with ∆SPT ≥ 20° with a p-value < 0.05. Data were checked for multicollinearity by using the Belsley-Kuh-Welsch technique. Heteroskedasticity and normality of residuals were assessed by the White test and the Shapiro-Wilk test, respectively. A p-value < 0.05 was considered statistically significant. All analyses were performed using R version 4.0.0 (R Foundation for Statistical Computing, Austria).

## Results

### Analysis of the LL shape in the overall cohort

The characteristics of the three groups of lumbar shapes are described in Table I.

**Table I.** Baseline characteristics of the patients in terms of lumbar shape.

Variable	Group 1: low PI/low lordosis, (n = 53; 17%)	Group 2 : low PI anteverted (n = 34; 11%)	Group 3 : high PI/high lordosis (n = 224; 72%)	p-value
**Baseline characteristics**				
Mean age, yrs (range)	65.1 (36 to 78)	61.7 (32 to 76)	63.7 (24 to 82)	0.627*
Females, n (%)	34 (64.1)	22 (64.7)	126 (56.2)	0.427†
**Mean spinopelvic parameters, ° (SD)**				
Pelvic incidence	42.1 (6.3)	42.9 (3.8)	60.5 (9.1)	< 0.001*
Lumbar lordosis	44.5 (10.2)	61.5 (11.0)	61.2 (10.4)	< 0.001‡
Standing SPT	-2.2 (7.3)	5 (7.8)	-0.1 (8.3)	< 0.001*
Sacral slope	29.8 (5.3)	41.6 (5)	44.9 (7.59)	< 0.001‡
Lumbar flexion	45.4 (12.6)	56.4 (14.1)	52.6 (13.6)	< 0.001‡
PI-LL mismatches	-2 (11.6)	-15.4 (10.7)	-0.7 (11.1)	< 0.001*
∆SPT	10.5 (15.6)	-0.15 (16.7)	0.8 (18.6)	0.002*
∆PFA	96.1 (18.4)	85.8 ( 17.4)	86.2 (20.8)	0.005*

* Kruskal-Wallis test.

† Chi-squared test.

‡ Analysis of variance (ANOVA) test.

LL, lumbar lordosis; PFA, pelvic femoral angle; PI, pelvic incidence; SD, standard deviation; SPT, spinopelvic tilt; SPT, spinopelvic tilt.

**Fig. 1:**
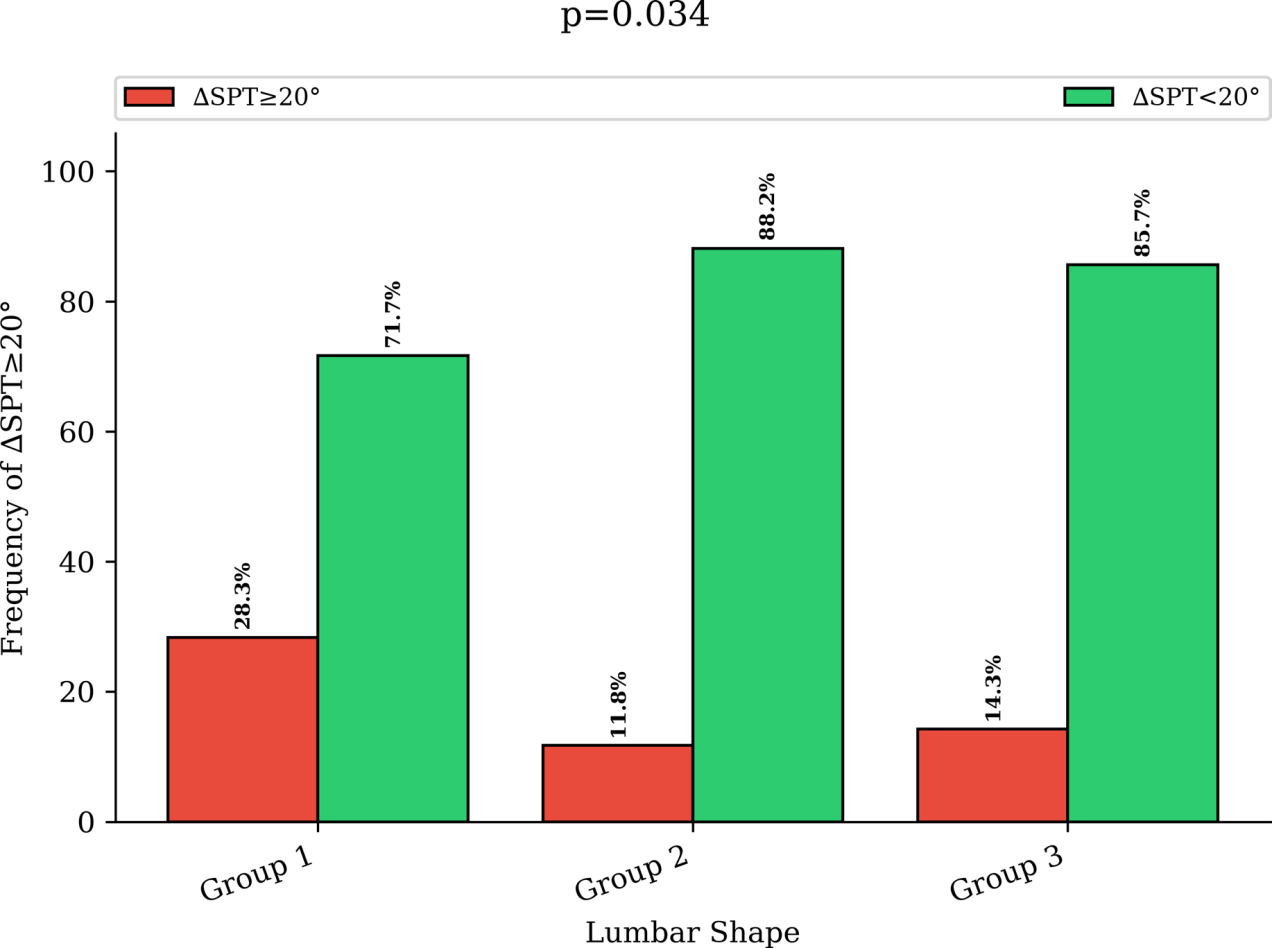
Groups of lumbar shape and association with abnormal spinopelvic mobility. This figure shows the risk of having abnormal spinopelvic mobility (∆SPT ≥ 20°) from the standing to sitting position in different groups of patients with variable lumbar shapes. SPT, spinopelvic tilt.

ΔSPT ≥ 20° rates were 28.3% (15/53), 11.8% (4/34), and 14.3% (32/224) for patients whose LL shape was associated with low PI low lordosis (group 1), low PI anteverted (group 2) and high PI/high lordosis (group 3), respectively (p = 0.034, chi-squared test) (Figure 1).

### Risk factors associated with abnormal spinopelvic mobility from the standing to flexed-sitting position

The rate of ∆SPT ≥ 20° in the overall population was 16.4% (51/311). The univariate analysis showed that the following predictors of ΔSPT ≥ 20° were IP-LL ≥ 20 (odds ratio (OR) 6.8 (95% confidence interval CI) 1.99 to 23.23), p = 0.004, Wald test), SPT≤-10° (OR 4.29 (95% CI2.05 to 8.99), p < 0.001, Wald test), LF ≤ 20° (OR 8.06 (95% CI 1.31 to 49.5), p = 0.033, Wald test), low PI/low lordosis (group 1) (OR 2.43 (95% CI 1.22 to 4.8), p = 0.014, Wald test), and SS < 35° (OR 2.71 (95% CI 1.43 to 5.14), p = 0.003, Wald test). There was no association between PI ≤ 41° and ΔSPT ≥ 20° (OR 2.01 (95% CI 0.88 to 4.62), p = 0.136, Wald test) and no association between group 2 (OR 0.65 (0.22 to 1.94), p = 0.624, Wald test), or group 3 (OR 0.6 (95% CI 0.32 to 1.12), p = 0.149, Wald test) and ∆SPT ≥ 20° (Table II).

**Table II.** Univariate analysis of the factors associated with ∆SPT ≥ 20°.

Variable	Patients, n	Patients with ∆SPT ≥ 20°, n (%)	Odds ratio (95% CI)	p-value*
**SPT ≤ -10°**				
No	273	36 (13.2)	1.0 (N/A)	
Yes	38	15 (39.4)	4.29 (2.05 to 8.99)	< 0.0001
**PI-LL ≥ 20**				
No	300	45 (15)	1.0 (N/A)	
Yes	11	6 (54.5)	6.8 (1.99 to 23.23)	0.004
**LF ≤ 20°**				
No	306	48 (15.7)	1.0 (N/A)	
Yes	5	3 (60)	8.06 (1.31 to 49.5)	0.033
**PI ≤ 41°**				
No	277	42 (15.2)	1.0 (N/A)	
Yes	35	8 (22.8)	2.01 (0.88 to 4.62)	0.136
**SS < 35°**				
No	241	31 (12.8)	1.0 (N/A)	
Yes	70	20 (28.5)	2.71 (1.43 to 5.14)	0.003
**Group 1**				
No	258	36 (13.9)	1.0 (N/A)	
Yes	53	15 (28.3)	2.43 (1.22 to 4.87)	0.014
**Group 2**				
No	277	47 (16.9)	1.0 (N/A)	
Yes	34	4 (11.8)	0.65 (0.22 to 1.94)	0.624
**Group 3**				
No	87	19 (21.8)	1.0 (N/A)	
Yes	224	32 (14.3)	0.6 (0.32 to 1.12)	0.149

* Univariate analysis.

CI, confidence interval; LF, lumbar flexion; LL, lumbar lordosis; N/A, not applicable; PI, pelvic incidence; SPT, spinopelvic tilt; SS, sacral slope.

**Fig. 2:**
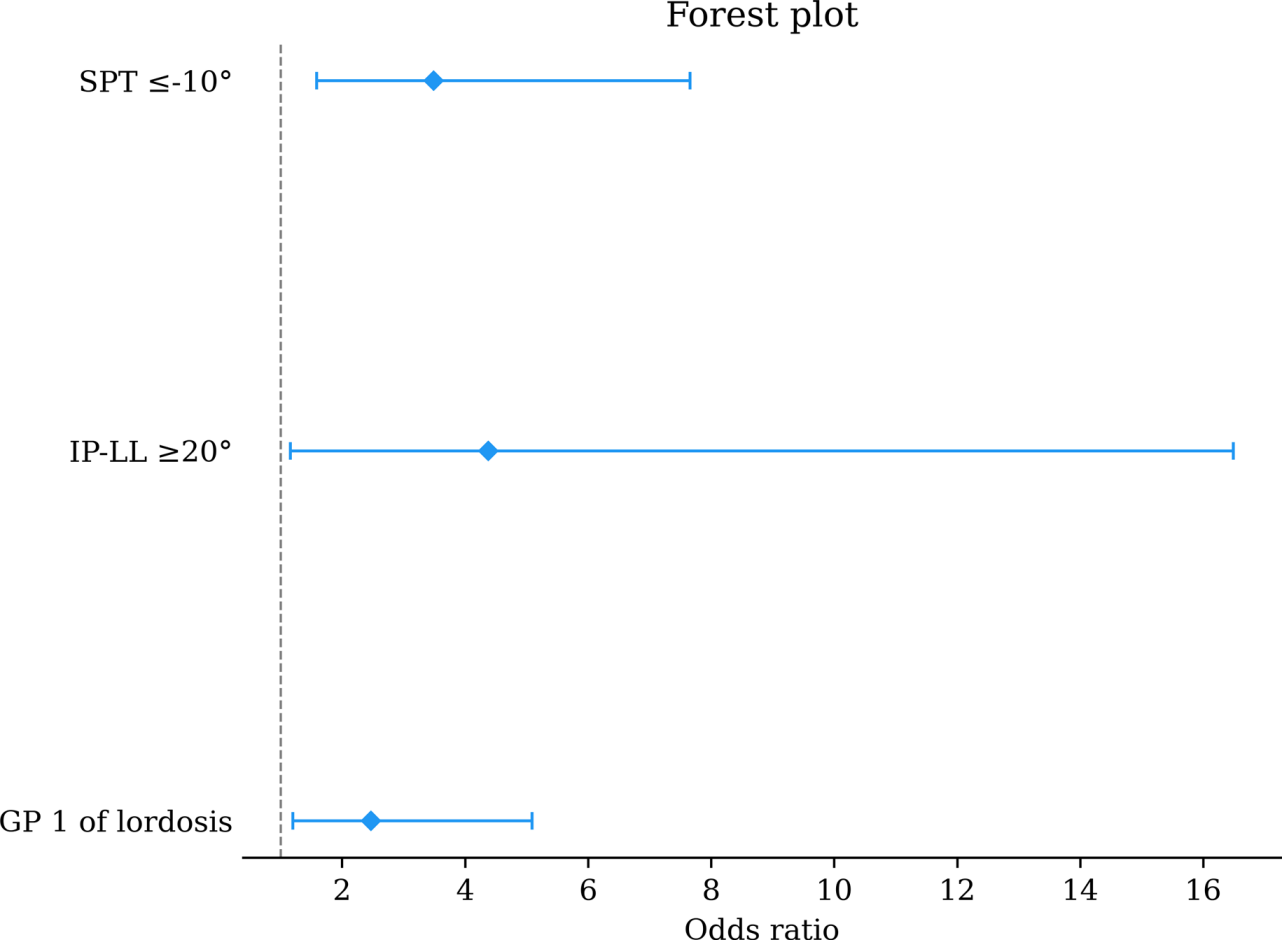
Factors independently associated with ∆SPT ≥ 20° in the multivariate multinomial logistic regression analysis. This figure shows the association between the variables that were included in the multivariate analysis. GP, group one; LL, lumbar lordosis; PI, pelvic incidence; SPT, spinopelvic tilt.

In the multivariate analysis, the following independent predictors of ΔSPT ≥ 20° were identified as SPT≤-10° (OR 3.49 (95% CI 1.59 to 7.66), p = 0.002, Wald test), IP-LL ≥ 20 (OR 4.38 (95% CI 1.16 to 16.48), p = 0.029, Wald test), and low PI/low lordosis (OR 2.47 (95% CI 1.19 to 5.09), p = 0.0148, Wald test) (Figure 2 and Table III).

**Table III.** Multivariate analysis of the factors associated with ∆SPT ≥ 20°.

Variable	Odds ratio (95% CI)	p-value*
SPT ≤ -10°	3.49 (1.59 to 7.66)	0.002
PI-LL ≥ 20°	4.38 (1.16 to 16.48)	0.029
Groupe 1 of lordosis	2.47 (1.19 to 5.09)	0.014

* Multivariate analysis.

CI, confidence interval; LF, lumbar flexion; LL, lumbar lordosis; PI-LL, pelvic incidence; SPT, spinopelvic tilt.

## Discussion

### Analysis of the different groups of lumbar lordosis

The first group corresponds to patients with a low PI and an apex of LL at the L5 level (Roussouly type 1) or the disk of L4/L5 (Roussouly type 2). These patients are identified as “hip users”,^4^ and have less flexion of the spine when sitting, which is described as a stuck pelvis in a relaxed-seated position^9,17^ or an abnormal hypermobile pelvis in a flexed-seated position.^13^ They had a mean anterior rotation of the pelvis of 10.5° and more flexion of the hip (∆PFA 96.1°).

In contrast, patients in group 3 with a high PI (> 45°) and higher lumbar lordosis with an apex of the LL at the L4 or L3 level (Roussouly type 3 and 4), represented a major part of the population (72%). They were identified as “spine users”^4^ and had less abnormal lumbopelvic cinematic and less flexion of the hip. The mean rotation of the pelvis when sitting was 0.8°, and the mean ∆PFA was 86.2°.

Finally, we isolated patients with a low PI (≤ 45°) and an apex of the LL at the L4 level and high lordosis in the group 2. These patients are described as type 3AP,^12^ and have a unusual spine shape, occurring in 11% of our population and represented more than a third of patients with a low PI. Except for the PI, they presented the same spinopelvic parameters with the same LL, LF, and ∆PFA as the “spine users”, as already published,^18^ and the same rotation of the pelvis when sitting (mean ∆SPT -0.15°). However, the PI-LL was lower (-15.4 to -0.7), which was explained by an unusual high lordosis but lower PI. Concerning abnormal spinopelvic mobility of the pelvis, the rates of ∆SPT ≥ 20° were comparable in groups 2 and 3 (11.8% vs 14.3%). This confirmed that PI alone is probably not a risk factor, but LL and its distribution are more likely to be risk factors.^18^ Patients with a low PI, lower lordosis, and a lower apex of LL have shown a significantly increased risk of ∆SPT ≥ 20°, with almost a third of patients being hypermobile (p <= 0.034) and having a higher risk of impingement and/or dislocation.^6^

### Comparison with other analyses of risk factors

In this study, we found that the variables that were statistically associated with the risk of abnormal spinopelvic mobility before THA were a standing SPT≤-10°, IP-LL ≥ 20°, and LF ≥ 20°, which matched the actual literature;^5,7,19^ however, if the univariate analysis showed that a stiff spine with an LF ≤ 20° was associated, it was not in the multivariate analysis. This result is probably due to the close relationship between SSD and spine stiffness. Low PI/low lordosis was the only characteristic that was associated with a risk of abnormal SPM, and was independently associated with ∆SPT ≥ 20° in the multivariate analysis. If a SS < 35° was associated with a higher risk in the univariate analysis, we omitted the variable because of multicollinearity between this variable and low PI/low lordosis because such variables are strongly dependent on each other (PI = SS-SPT).^12^

PI alone was not associated with a higher risk of having an abnormal SPM, thereby confirming the findings of recent studies.^6,7^ Regardless of a patient’s PI, having a SSD, either degenerative or iatrogenic, and analyzing the implication of PI with these patients could be a confounding factor. Nevertheless, patients with a lower PI in our study had a higher rate of ∆SPT ≥ 20°, 23%, and only 11% in a large study of more than 9,000 patients, with a PI ≤ 41° have abnormal spinopelvic mobility.^7^

**Fig. 3:**
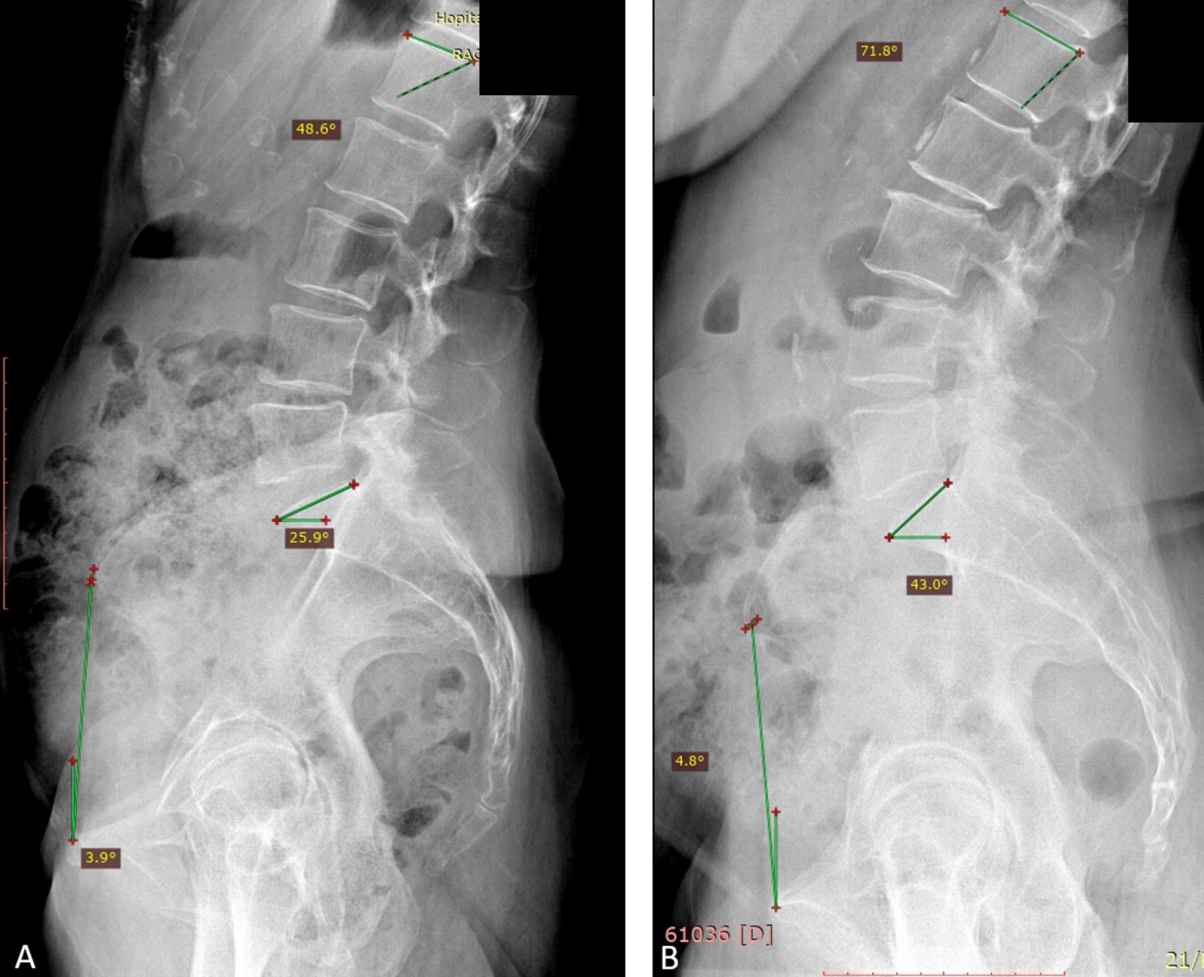
Type of lumbar shape in patients with low pelvic incidence (PI) in lateral standing radiographs. a) Patient with low PI and low lordosis. PI = 38°, spinopelvic tilt (SPT) = -4°, lumbar lordosis (LL) 49°, sacral slope (SS) 26°, and lumbar apex at level L5. b) Patient with low anteverted PI 39°, SPT 5°, LL 71°, SS 42°, and lumbar apex at level L4.

This result supports the findings of studies that analyzed the clinical data of patients with low lordosis who had an increased risk of anterior impingement.^9,20^ Patients with both low PI and low LL were analyzed. They ultimately concluded that PI was a key factor in patients who underwent THA. The physiology of this type of back (Roussouly type 3 AP) divulges the association among low PI, high LL, and high sacral slope, and explains why, in a large cohort of patients, a low PI is not associated with a higher risk of having abnormal spinopelvic mobility.^10^ If PI remains unchanged over the life course, this shape is also possible by the arithmetic formula PI = SS-SPT because of a higher SPT (5.8° in our study) is associated with a higher SS (41.3°) (Figure 3).

The rate of ∆SPT ≥ 20° in patients without an SSD was 15%, corresponding to 45 patients. It seems that considering only patients with a severe sagittal spinal deformity, excessive standing posterior spinopelvic tilt, or lumbar spine stiffness is not enough to identify all patients with a risk of impingement or dislocation. Patients identified as “hip users” with low lordosis (group 1) should be investigated to even more precisely detect patients who require a modified position of the implant and/or a more constraint bearing to avoid low functional outcomes.

The present study has certain limitations. First, we retrospectively analyzed a consecutive cohort; prospective validation remains desirable. Second, the classification of the shape of the LL was conducted in a healthy young population without any lumbar diseases or low back pain.^12^ However, we did not analyze the association of lumbar diseases and spinopelvic mobility. Nevertheless, the different types of LL are predisposed to different degenerative changes:^21,22^ low PI with a distal apex of the LL is associated with higher axial stress from L4-L5 and L5-S1 discs and with spondylolisthesis at the level L5-S1, whereas higher lordosis with a lumbar apex at level L4 is less predisposed to degenerative pathology. A recent study showed the association between a narrowed disc space involving the L5-S1 segment and spinopelvic motion in patients undergoing THA,^23^ which could be explained by the association of a low PI, low lordosis, and an apex of LL at a distal level.

Third, even if the Roussouly classification has shown good reliability and reproducibility,^24^ patients with borderline cut-off values of SS often have different measurements, ≤ 35° in the distal apex of lordosis, and between 35 and 45° in anteverted patients. However, the SS measurement did correlate with these borderlines, with 29.9° (standard deviation (SD) 5°) for the former group and 41.3° (SD 5°) for the latter. The definition of the lordotic apex may be doubtful, allowing divergences between types 1 and 2.^24^ Nevertheless, we analyzed patients with a distal apex of lordosis together, patient with an apex at L5-S1 progress to eventually have an apex at the L5 level when ageing,^25^ and both have shown the same biomechanics.^18^ However, it might be possible that this analysis underestimates the rate of patients with a low anteverted PI, corresponding to 16% of the population in other studies of healthy patients (vs 11% in our cohort),^12^ but it is the first analysis of this spine shape in patients suffering from hip arthritis. Furthermore, we only used PI-LL mismatch for the distinction between patients with and without SSD, and we acknowledge that it could have caused bias without a global analysis of the sagittal balance,^26^ or an individual medical history of fracture or surgery.

Finally, we did not analyze the risk of impingement or dislocation in this retrospective study, but only the rate of abnormal SPM. Instability and impingement are associated with other factors, including implant positions, combined anteversion, leg length, and offset,^27-29^ and should be anticipated before surgery. The purpose was only to understand the implication of a low PI in patients with abnormal spinopelvic mobility.

In conclusion, if the PI value alone is not indicative of spinopelvic mobility, patients with a low PI, low lordosis, and lumbar apex at the L5 or L4-L5 level (Roussouly type I and II) had higher rates of having abnormal spinopelvic mobility from standing to sitting than patients with low PI anteverted with high lordosis (Roussouly type III anteverted). The findings should be analyzed independently.

**Take home message**

- A low pelvic incidence (PI) associated with low lordosis is associated with higher rates of abnormal spinopelvic mobility (∆SPT ≥ 20°).

- A low PI can be associated with high lordosis and does not adversely affect spinopelvic mobility.

- Patient with low lordosis have a lumbar apex at L4-L5 or below.
